# Connectome topology of mammalian brains and its relationship to taxonomy and phylogeny

**DOI:** 10.3389/fnins.2022.1044372

**Published:** 2023-01-11

**Authors:** Joshua Faskowitz, Maria Grazia Puxeddu, Martijn P. van den Heuvel, Bratislav Mišić, Yossi Yovel, Yaniv Assaf, Richard F. Betzel, Olaf Sporns

**Affiliations:** ^1^Department of Psychological and Brain Sciences, Indiana University Bloomington, Bloomington, IN, United States; ^2^Department of Complex Trait Genetics, Center for Neurogenomics and Cognitive Research, Vrije Universiteit Amsterdam, Amsterdam, Netherlands; ^3^Montreal Neurological Institute, McGill University, Montreal, QC, Canada; ^4^School of Neurobiology, Biochemistry and Biophysics, Tel Aviv University, Tel Aviv-Yafo, Israel; ^5^Program in Neuroscience, Indiana University Bloomington, Bloomington, IN, United States; ^6^Program in Cognitive Science, Indiana University Bloomington, Bloomington, IN, United States; ^7^Indiana University Network Science Institute, Indiana University Bloomington, Bloomington, IN, United States

**Keywords:** network neuroscience, connectomics, mammals, phylogeny, comparative neuroanatomy, connectome analysis

## Abstract

Network models of anatomical connections allow for the extraction of quantitative features describing brain organization, and their comparison across brains from different species. Such comparisons can inform our understanding of between-species differences in brain architecture and can be compared to existing taxonomies and phylogenies. Here we performed a quantitative comparative analysis using the MaMI database (Tel Aviv University), a collection of brain networks reconstructed from *ex vivo* diffusion MRI spanning 125 species and 12 taxonomic orders or superorders. We used a broad range of metrics to measure between-mammal distances and compare these estimates to the separation of species as derived from taxonomy and phylogeny. We found that within-taxonomy order network distances are significantly closer than between-taxonomy network distances, and this relation holds for several measures of network distance. Furthermore, to estimate the evolutionary divergence between species, we obtained phylogenetic distances across 10,000 plausible phylogenetic trees. The anatomical network distances were rank-correlated with phylogenetic distances 10,000 times, creating a distribution of coefficients that demonstrate significantly positive correlations between network and phylogenetic distances. Collectively, these analyses demonstrate species-level organization across scales and informational sources: we relate brain networks distances, derived from MRI, with evolutionary distances, derived from genotyping data.

## Introduction

Mammals come in a wide range of morphologies, with each of roughly 6,000 species adapted to the diversity of the Earth’s ecosystems. Accordingly, the brains of mammals vary in shapes, sizes, and capacities to support different kinds of cognition and behavior. Despite this variation in overall form and ecological adaptations, all mammalian brains are organized into networks that form complex webs of interacting neurons supporting signaling and communication (Laughlin and Sejnowski, 2003; Sporns et al., 2004). The principles that govern common features of network architecture across species, as well as those feature patterns that differentiate them, have the potential to shed light on the link between neuroanatomy and function (Van den Heuvel et al., 2016; Sousa et al., 2017; Ardesch et al., 2019; DeCasien et al., 2022). Across species we can consider quantitative markers of brain shape, cytoarchitectonics, and histology to search for relationships that might illuminate the evolutionary constraints placed on brain morphology. Along these lines, features such as the ratio of gray matter to white matter (Zhang and Sejnowski, 2000) or combinations of several features, such as gyrification, neuronal density, and neuronal tissue volumes (Herculano-Houzel et al., 2010; Mota et al., 2019) have been shown to exhibit (often allometric) scaling relations with brain size or volume. Another way in which to evaluate the imprint of evolutionary constraints on brain architecture is to comparatively study the networked pattern of neural wiring.

The mammalian brain can be conceptualized as a network of interconnected components—an abstraction that allows for the extraction of relevant features describing local and global brain organization (Bassett and Sporns, 2017). Network modeling allows for the wiring patterns of the brain to be quantitatively characterized, making topological patterns accessible to comparative analysis. Using such an approach, the wiring cost of different mammals has been assessed (Cherniak, 1994; Kaiser and Hilgetag, 2006; Rivera-Alba et al., 2011), suggesting a tradeoff between conservation of neural resources and the promotion of neural communication (Bullmore and Sporns, 2012; Van den Heuvel et al., 2016). Brain networks have also been used to study how neuroanatomy scales with size. A recent analysis of 14 primate species demonstrated that the propensity for short connections to form was positively correlated with brain volume, along with network properties such as the clustering coefficient and characteristic path length (Ardesch et al., 2021).

Several approaches to comparative neuroanatomy studies involve the search for homologous connectivity patterns (Balsters et al., 2020; Roumazeilles et al., 2022), examine specific regions or systems for connectional differences (Rilling et al., 2008; Rilling, 2014), or involve the creation of surface maps that reveal inter-species relationships in regional size and differentiation (Van Essen et al., 2019; Xu et al., 2020). Common to these approaches is that pattern identification must rely on pre-identified regions and/or a diffeomorphic transformation between neuroanatomical maps. While this is less of a barrier for studies comparing species with similar brain architectures and established homology relations (e.g., primates), it does present significant difficulties for comparing vastly different mammals for which detailed areal maps are not available, like a bat to a dolphin or a zebra to a vole. Comparative studies that span multiple taxonomic clades require an approach that is agnostic to neuroanatomical homologies. One candidate approach is network modeling.

**FIGURE 1:**
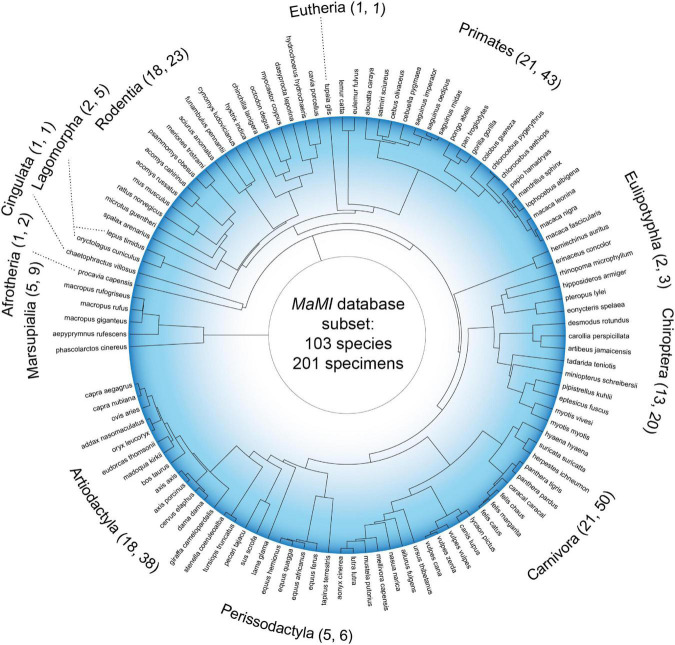
Phylogenetic tree of the mammals under investigation. Tree distances are derived from a consensus phylogeny estimate (Upham et al., 2019); 103 species names and 12 taxonomic orders are shown. For each taxonomic order label, the first number in parenthesis denotes the number of unique species represented in the underlying tree branch (which sums to 103), whereas the second number denotes the number of specimens for each order (which sums to 201).

In the present study, we take advantage of the recently published MaMI (Mammalian MRI) database, a collection of brain networks derived from hundreds of mammals, using high-resolution *ex vivo* structural and diffusion magnetic resonance imaging (Assaf et al., 2020; Suárez et al., 2022). The mammals used for analyses here span 103 unique species and cover 12 taxonomic orders (Figure 1), capturing the richness of brain organization in mammals adapted to a wealth of ecologies. Using this data, it has already been shown that local graph features like the average clustering coefficient, betweenness centrality, and closeness centrality vary across taxonomic orders (Suárez et al., 2022). Yet across species, key topological factors such as the proportion of short, medium, and long connections are relatively consistent across species. Furthermore, it has been shown that the mean shortest path length, a purported marker of theoretical communication efficiency in brain networks, scales across species (Assaf et al., 2020). In this path-based analysis, it was proposed that intra-hemispheric connectivity is modulated by the number of commissural fibers to maintain similar overall connectivity across species. Collectively, these comparative network analyses emphasize that the topology of brain networks can serve to differentiate mammals, even if other network features remain constant.

Here, we seek to compare brain networks across mammalian species, to demonstrate how differences in brain architecture relate to mammalian classification. The quest to explore such a question is enriched by the diversity of the MaMI database, which provides examples of brains from several taxonomic orders. Previous work has initially demonstrated that brain networks within taxonomic order are less distant than between orders (Suárez et al., 2022). Here, we reproduce this finding, using a suite of network distance measures. We build upon this idea and extend previous work by asking if the pattern of brain network distances between mammals is similar to the estimated phylogenetic distances between mammals (Upham et al., 2019). In additional analyses, we account for differences in scanner signal-to-noise ratio, brain volume, and the spatial embeddedness of the brains when relating network and phylogenetic distances. Finally, we describe the differences between phylogenetic trees that correspond, strongly vs. weakly, to the brain network distances.

## Materials and methods

### Image acquisition

For this study, we used data from the mammalian MRI database (MaMI), a collection of 225 *ex vivo* diffusion weighted brain scans (Assaf et al., 2020; Suárez et al., 2022). These scans include 125 unique species and cover 12 taxonomic orders (clades): *Cingulata*, *Afrotheria*, *Artiodactyla*, *Perissodactyla*, *Carnivora*, *Chiroptera*, *Eulipotyphla*, *Primates*, *Eutheria*, *Rodentia*, *Lagomorpha*, *Marsupialia*. Detailed image acquisition protocols can be found in Assaf et al. (2020). No animals were deliberately euthanized for the present study. All brains were collected based on incidental death of animals in zoos in Israel or natural death collected in the wild and with the permission of the national park authority (approval no. 2012/38645), or its equivalent in the relevant countries. Specimen brains were extracted from the skull and fixated before scanning. Approximately 24 h before undergoing scanning, each brain was placed in phosphate-buffered saline for rehydration. Brains in the dataset differed in shape and size, necessitating different imaging equipment due to scanning bore limitations. Smaller brain (up to approximately 0.15 ml) images were collected on a 7 Tesla 30/70 Biospec Avance Bruker system. Larger brains (approximately >1000 ml) were collected on a 3 Tesla Siemens Prisma system. To reduce magnetic susceptibility artifacts, brains were scanned while immersed in fluorinated oil (Fluorine, 3M). Diffusion MRI data were acquired using high angular resolution diffusion imaging (HARDI), which consists of a series of diffusion-weighted, spin-echo, echo-planar-imaging images of the whole brain field of view. Imaging parameters for the 7T scanning acquisitions include: 60 gradient directions, 3 B0 volumes, *B*-value = 1000 s/mm^2^, TR > 12000 ms (depending on the number of slices), TE = 20 ms, big delta/little delta = 10/4.5 ms. Imaging parameters for the 3T scanning acquisitions include: 64 gradient directions, 3 B0 volumes, *B*-value = 1000 s/mm^2^, TR = 3500 ms, TE = 47 ms, big delta/little delta = 17/23 ms. Acquisition parameters were adjusted on a per-animal basis so that pixel size was linearly scaled across brains, ensuring that all scans were acquired with the same 2-dimensional pixel grid (128 × 96). Based on brain size and shape, the number of image slices varied between 46 and 68. An attempt was made to keep signal-to-noise ratio above 20 for all brains. This resulted in a difference in acquisition times, which approximated 48 h for small brains and 25 min for large brains. Several validations on the effect of varying scanning parameters were performed in Assaf et al. (2020). In this previous study the authors demonstrated that the collected data correlated strongly with externally acquired mouse data. These analyses support the claim that the differing scan parameters were sensibly applied, and that cross-species acquisition biases have been addressed to the extent possible.

### Brain network construction

Diffusion weighted data was pre-processed using ExploreDTI (Leemans et al., 2009). Pre-processing included anisotropic smoothing of image slices with a 3-pixel Gaussian kernel, as well as motion, susceptibility, and eddy current distortion correction. Fiber orientation direction was estimated using the spherical deconvolution approach (Tournier et al., 2004), allowing for the modeling of crossing fiber orientations in each voxel. The maximum spherical harmonic order applied was four. Following fiber orientation modeling, whole brain tractography was performed using a seed point threshold of 0.2 and a half-pixel step length. This tractography method ensures that approximately 90% of the streamline endpoints terminate in the cortical and subcortical gray matter. Streamlines passing through the cerebral peduncle and cerebellar connections were filtered out of the tractography before network reconstruction.

A standard parcellation procedure was applied to the tractography data of each mammal, to create an equal number of nodes for each brain. For each image, the brain’s midline was manually identified. For each hemisphere, streamline endpoints in Euclidean space were parcellated into 100 regions using k-means clustering (as implemented in MATLAB), considering all fiber endpoints positions. Therefore, endpoints with similar spatial termination patterns were more likely to be assigned to the same cluster. Volumetric nodes were taken to be the center of mass of the resultant 200 k-means-identified non-overlapping regions, with 100 nodes per hemisphere. Note that the parcellation strategy employed here differs from a random partition of cortical volumes (Zalesky et al., 2010) or surfaces (Ardesch et al., 2021). Structural connectivity weights were taken to be the count of streamlines connecting volumetric nodes, divided by the combined geometric mean volume of these nodes (Faskowitz et al., 2018). Fiber length was taken to be the average length of all the streamlines connecting nodes. The Euclidean distance between nodes was taken to be the straight-line distance between the center of mass of volumetric nodes. Euclidean distances were computed in voxel units as opposed to real-world units.

The parcellation approach described here can be repeated using different initial conditions of the k-means clustering algorithm, rendering slightly different 200-node parcellations and resultant structural networks. For each mammal, 100 runs of the parcellation procedure were carried out, resulting in 100 instantiations of the brain network for each animal. Each such set represents an ensemble of plausible network representations derived from the same underlying tractography data.

### Network comparison without node correspondence

Common practice in network neuroscience is to use a parcellation in either stereotaxic space or fit to native space to define network nodes (Eickhoff et al., 2018). Such an approach allows for a straightforward comparison of individual networks, as it is assumed that nodes correspond to the same structural or functional regions across brains. In this case, brain networks can be compared with straightforward operations such as the correlation of two matrices’ upper-triangles, assuming networks are undirected (Finn et al., 2015). In this study, due to the heterogeneity of brains included in the database, a common parcellation approach is not feasible (Ardesch et al., 2021). Therefore, comparing brain networks necessitated a network comparison methodology that does not require nodal correspondence across brains. For this, we opted to use the network portrait divergence (Bagrow and Bollt, 2019).

A network portrait is a representation of a network’s geodesic, or shortest-path, distance distribution (Bagrow et al., 2008). Briefly, a network portrait *B* can be constructed on an unweighted network by taking a histogram of a binary matrix thresholded at each shortest path length *l* ∈ {0,…,*L*} where *L* is the diameter of the network, and then stacking these histograms to form a rectangular matrix *B*, where the entry *B*_*l,k*_ encodes the number of nodes, *n*, who have *k* nodes at distance *l*. For weighted networks, continuous distances need to be discretized into bins, using a number of bins chosen *a priori* or based on a heuristic histogram rule. The network portrait divergence is computed by taking the Jensen–Shannon divergence between network portraits. This approach can accommodate the comparison of differently sized networks and does not necessarily require node correspondence. The utilization of this distance measure was motivated by the shortest path-based analyses conducted previously on these data, which noted that mean shortest path distance is weakly related to brain volume, displaying merely a 40% linear increase over four orders of magnitude of brain volumes (Assaf et al., 2020). Furthermore, this study demonstrated a robust link between intra-hemispheric, mean shortest path distance, and commissural ratio percentage, which persisted across parcellation size and scanning resolution. A method based on shortest paths was chosen as the primary network comparison tool based on these previous findings demonstrating feature stability. Shortest paths on structural brain networks can be interpreted as a proxy for polysynaptic signaling capacity between regions (Avena-Koenigsberger et al., 2018; Vázquez-Rodriguez et al., 2020). Alternative comparison tools were also employed, to demonstrate robustness (see below).

In the present study, network portraits were constructed using 25 quantiles to discretize the continuous geodesic distances. Bin size was held constant, as opposed to using a binning heuristic, so that comparisons between all mammals are comparable. Continuous geodesic distances were computed by taking the inverse of structural edges to be between-node distances and using the Brain Connectivity Toolbox (BCT) (Rubinov and Sporns, 2010) function *distance_wei_floyd* (Floyd, 1962).

### Density-based thresholding

To ensure that reported results were not due to variation in structural network density, which can influence common brain network analysis measures (Van Wijk et al., 2010), alternative versions of each mammalian brain network were constructed after thresholding each network at a constant density *d* = {0.05, 0.10, 0.15}. That is, for each density *d*, first the maximum spanning tree was computed, retaining a skeleton of the *N-1* largest edge magnitudes that form a connected component. Then, edges not included in this skeleton are added in order of descending edge weight, until a density of *d* for the network is met. Note that the original edge weights were retained for the supra-threshold edges. This procedure ensures that thresholding does not fragment the network into disconnected components (unless the original network is not a single connected component to begin with).

### Centroid extraction and outlier removal

Mammalian brain networks were compared both within and between mammals using the network portrait divergence to identify excessively distant networks. First, for each mammal, its 100 network instantiations were compared in a pairwise manner, forming a 100-by-100 distance matrix. The rows of this matrix were averaged, forming a list of average distances from each instantiation to all other instantiations. Average distance values greater than 3 scaled median absolute deviations (MAD) from the median, identified with MATLAB function *isoutlier*, were marked as outlier networks for removal. Of the remaining brain networks, the network with the minimal average distance to other instantiations was retained as the representative, or centroid, network. Mammalian centroid brain networks were compared to each other in a pairwise manner, forming a 225-by-225 distance matrix. The rows of this matrix were averaged, forming a list of average distances from each instantiation to all other instantiations. Average distance values more than 3 MAD from the median were marked as outliers. Using the 12 taxonomic orders to define modules on the 225-by-225 distance matrix, we computed the within-module degree z-score. Nodes (i.e., mammals) that had within-module degree z-scores greater than 3 were additionally marked as outliers, as this measure would indicate the magnitude of deviation of distance values within each module (see Supplementary Figure 1 for a visualization of the differences between retained and outlier data). These procedures for centroid extraction and outlier removal were repeated separately for the non-thresholded and the three thresholded versions of the data, creating four sets of mammalian brain data for analysis. These sets of data contain *N* = {201, 201, 201, 190} mammals, at thresholds of *d* = {*none*, 0.05, 0.10, 0.15} respectively. The main text includes analyses with the *d* = 0.10 dataset (Supplementary Figure 6) so as to report findings that are not biased by varying network densities (Van Wijk et al., 2010); analyses performed at alternative thresholds *d* = {*none*, 0.05, 0.15} are reported in Supplementary Information.

### Alternative network comparison methods

Additional network comparison operations were computed with a suite of methods that do not require node correspondence (Tantardini et al., 2019; Mheich et al., 2020; Wills and Meyer, 2020).

### Generative model distance

One way to perform network comparison is to derive topological descriptors from each network and in turn, compare these measurements or statistics to each other. Such an approach abstracts a network into a measurement distribution or set of central tendency measures that can be compared in a standardized way, even if the number of nodes differs between networks. In Betzel et al. (2016), the distance between networks was taken to be the maximum Kolmogorov-Smirnov (KS) distance across the comparisons of the binary degree, binary clustering coefficient, binary betweenness, and edge length distributions. This distance can also be assessed as the mean KS of these distributions (Faskowitz et al., 2018) and can be assessed using the weighted variants of each topological measurement.

### NetSimile

The NetSimile method utilizes topological and ego-network (egonet) concepts to compare networks of different sizes (Berlingerio et al., 2012), where an egonet is defined by the induced subgraph of a node and its neighbors. The method requires the extraction of seven node-wise distributions for each network: degree, clustering coefficient, average degree of 2-hop neighbors, average clustering coefficient of neighbors, average egonet clustering coefficient, weight of egonet outgoing edges, and summed degree of each egonet. From these seven distributions, five features are taken from each distribution (median, mean, standard deviation, skewness, and kurtosis) forming a “signature” vector of each network of length 35. Signature vectors are then compared by taking the Canberra distance between them. As with the generative modeling distance, weighted variants of the topological measures can be taken.

### Laplacian and adjacency spectral distances

Spectral distances between graphs are taken by comparing a sequence of eigenvalues derived from each network (de Lange et al., 2014; Dodonova et al., 2016; Kurmukov et al., 2016; Wills and Meyer, 2020). To take a spectral distance, first, an eigen-decomposition is performed on either the network’s Laplacian or adjacency matrix, sorted in order of magnitude, and a predetermined *k* number of eigenvalues are retained for comparison. For comparisons of the Laplacian-derived eigenvalues, the *k* smallest values are retained, whereas for comparisons of the adjacency-derived eigenvalues, the *k* largest eigenvalues are retained. Comparisons can be made using the full eigen-spectrum but are commonly taken when *k* ≪ *n*, where n is the number of nodes in a network (Wills and Meyer, 2020). The spectral distance can be taken as the Euclidean distance between the spectra of length *k*. This method allows for the comparison of differently sized networks without node correspondence, if *k* is less than or equal to the number of nodes in each network. In the current study, spectral distances were taken by measuring the *l_2_* metric (i.e., Euclidean distance) between the spectrums at *k* = {5, 50, 100,200} for both adjacency and Laplacian-derived spectra. Note, when comparing networks with disconnected nodes, the full spectra comparison *k* = 200, was reduced to match the size of the largest connected component of the smaller network (i.e., *k* = 199, if one node was found to be disconnected). Finally, an additional distance measure was calculated by taking the Jensen–Shannon divergence between the density-normalized Laplacian spectra (De Domenico and Biamonte, 2016).

### Phylogenetic distance estimation

Phylogenetic distances were estimated using a set of 10,000 complete phylogenetic trees inferred using a Bayesian approach (Upham et al., 2019). Using a large set of plausible trees, rather than a single consensus tree, allows for a better characterization of the uncertainty associated with tree estimation (Huelsenbeck et al., 2000). For each complete tree, pairwise distances between species were computed by adding patristic distance along tree branches using MATLAB’s pdist (phytree) function. Distance matrices were then pruned to match the composition of mammalian species contained within the MaMI database.

### Generative model parameter estimation

For each mammalian network we sought to estimate the influence that space has on the formation of edges between nodes, which might vary across species. After estimating such a parameter, it could be used to as a covariate of no interest (i.e., nuisance regressor) to account for differences in spatial embedding. To operationalize spatial embeddedness, we estimated a spatial wiring rule as a power-law function (Vertes et al., 2012; Betzel et al., 2016) following the formula:


$$P\,\left(u,v\right)=E\,{\left(u,v\right)}^{\eta }$$

where the probability of forming a connection between nodes *u* and *v*, *P*(*u*,*v*), is given by the Euclidean distance between the nodes *E*(*u*,*v*) raised to a free parameter, η. This parameter can be conceptualized as quantifying the distance penalty (negative setting for the exponent η) incurred as connections increase in length. To estimate η for each network, the maximum spanning tree was taken as a seed set of edges. The η parameter was uniformly sampled 200 times between the range −15 to 0 and for each candidate parameter, a corresponding binary synthetic network was generated. Synthetic networks were evaluated against the original network using the binary generative modeling distance (maximum KS) described previously. The least distant 10% of synthetic networks were retained. Additionally, the bottom 20% of synthetic networks were randomly sampled according to a probability proportional to the inverse of network distance. The overlap of these samples was used to gather candidate η parameters. The minimum and maximum of this set formed new lower and upper bounds defining a range from which one can sample uniformly. This process was carried out five times, to identify the value of the η parameter that is least distant from the original network. After five iterations, a network’s η parameter was taken to be the median value from the 20% of least distant (i.e., lowest energy) synthetic networks. It should be noted that in this analysis, the generated matrices are binary, meaning that only edge-existence contributed to the estimation of the spatial embeddedness.

Following previous work, from which our code is adapted (Betzel et al., 2016), we elected to use a power-law relationship to operationalize spatial embeddedness. Alternative formulations capturing the role of spatial distance in wiring probability and density have been used in prior literature. For example, brain network wiring can also be modeled using an exponential distance rule, as has been employed in studies of mice (Oh et al., 2014; Horvat et al., 2016), marmosets (Theodoni et al., 2022), and macaques (Ercsey-Ravasz et al., 2013). To examine the impact of using an exponential decay function in place of a power law relationship, we have run the same generative modeling procedure using an exponential function.

## Results

Here we describe how distances between anatomical networks reflect taxonomic and phylogenetic differences between mammals. To achieve this, we computed the pairwise distances between the brain networks of various mammals. These distances, reflecting differences in neuroanatomical topological patterns, can be related to divisions in mammalian taxonomy and distances derived from phylogenetic trees.

### Network distances concur with taxonomic orders

**FIGURE 2:**
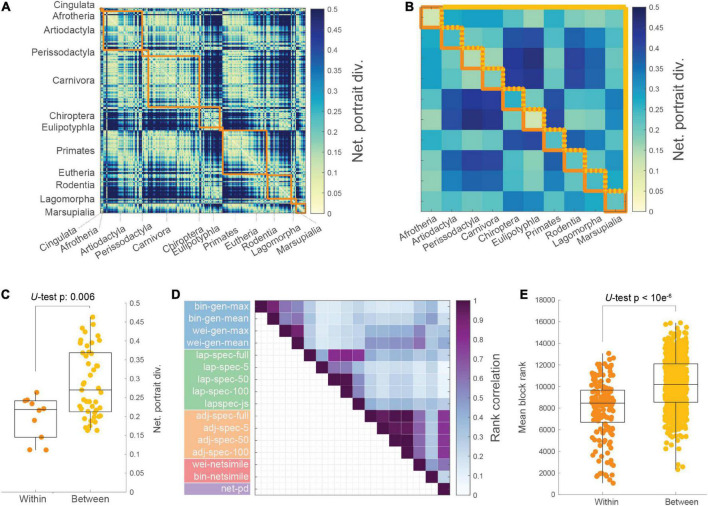
Network distances correspond to taxonomic order. **(A)** Network portrait divergence matrix (with dimensions 201-by-201) for all mammals, organized by taxonomy into on-diagonal blocks (orange). **(B)** Average network portrait divergence within (orange) and between (yellow) taxonomic blocks. **(C)** Within-order average distance is lower than between-order average distance (Mann-Whitney *U*-test, *p* = 0.006). **(D)** Rank correlations between distance patterns produced by a variety of distance measures; bin: binary; wei: weighted; lap: Laplacian matrix; adj: adjacency matrix; gen: generative distance; spec: spectral distance; js: Jensen–Shannon divergence; netsimile: NetSimile distance; net-pd: network portrait divergence. **(E)** Aggregating all distance measures, the ranked within-order distance is lower than the ranked between-order distance (*U*-test, *p* < 10^6^).

The pairwise network portrait divergence was taken between each mammalian structural brain network (Figure 2A). These distances were averaged within blocks formed by the 12 taxonomic orders of mammals covered in this sample (Figure 2B). For the purposes of within- and between-block comparisons, the orders *Cingulata* and *Eutheria* were excluded as the current version of the mammalian database contains just one animal in each order. Between-block distances were greater on average than within-block distances (Figure 2C; Mann-Whitney *U*-test, *p* = 0.006). Between-network distances were taken with alternative measures as well. Fifteen alternative measures were applied, falling into four categories: network feature histogram distances, Laplacian spectral distances, adjacency spectral distances, and NetSimile. For each measure, pairwise distances were calculated for each mammalian brain network, and then rank correlated with each other (Figure 2D; see Supplementary Table 1 for expansion of distance measure names). This visualization demonstrates the similarity between measures, which is largely organized by conceptual class (as indicated by colors underlying the measure names in Figure 2D). From this matrix, similarities outside of conceptual class, such as between network portrait divergence and the adjacency spectral distances, are also observable. For comparing between- and within-block distance magnitudes across measures, the distance matrix for each measure was rank transformed and then compared, showing that between-block distances were greater on average than within-block distances (Figure 2E; Mann-Whitney *U*-test, *p* < 10^–6^). In Supplementary Figure 2, these within- and between-block distances are shown with alternative thresholds, and in Supplementary Figure 3, the distances from each of the alternative methods are shown distinctly.

### Network distances relate to phylogenetic distances

Here we ask how the pairwise network distances relate to pairwise phylogenetic distances between mammals. In other words, we ask if the pattern of interrelationships between brain networks is like the pattern of interrelationships estimated from evolutionary history.

**FIGURE 3:**
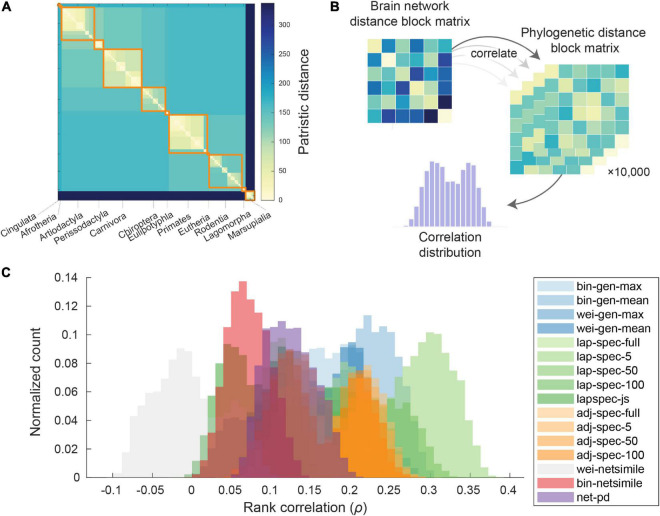
Network distance correlates with phylogenetic distance. **(A)** Phylogenetic distance matrix (with dimensions 103-by-103) for all unique mammal species, with taxonomy highlighted *via* orange on-diagonal blocks. **(B)** Schematic of the process for correlating brain network distance with multiple phylogenetic distance estimates. **(C)** For each distance measure, the distribution of Spearman rank correlation coefficients is shown for 10^3^ comparisons of block network distance to block phylogenetic distance; distributions are only colored if the bottom 1% correlation value exceeds zero; distributions are colored by distance measure category.

The pairwise patristic distance was determined from 10,000 estimated trees provided as a reference data set by Upham et al. (2019). The patristic distances were computed by summing the traversed distance on the tree, between each mammal. From Upham et al. (2019), a maximum clade credibility consensus tree was downloaded (at the link)^1^ and used for display purposes (Figure 3A) and to determine the taxonomy display ordering. For each network distance measure, distances within each taxonomic block were averaged; similarly, for each of the 10,000 phylogenetic trees, distances within each taxonomic block were averaged. These block matrices were then rank correlated (Figure 3B) and visualized as distributions of Spearman (rank) correlation coefficient distributions (Figure 3C). Distributions are colored by network distance measure category and were only colored if the lowest 1% distribution value exceeded zero. Only one measure, the weighted NetSimile distance, displayed a distribution of correlation values that was mostly negative. All other network distance measures had distributions with significantly positive correlation coefficients. Measures from the Laplacian spectral distance category display distributions with generally large magnitudes; the Laplacian distance using five dimensions achieved the highest correlation magnitudes. In Supplementary Figure 4, these relationships are visualized as two-dimensional histograms depicting the density of points across all 10,000 correlations.

**FIGURE 4:**
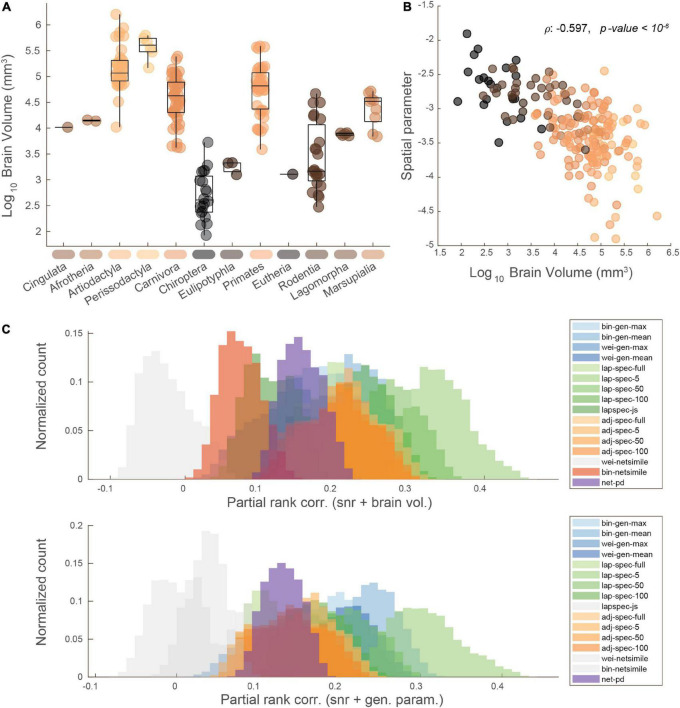
Accounting for potential confounds in network vs. phylogeny distance correlations. **(A)** Mammals log_10_ brain volume for each order; points colored by brain volume magnitude. **(B)** Brain volume plotted against a generative spatial parameter, showing how larger brains’ connectivity patterns are more influenced (more negative) by the distance between connections; points colored by brain volume magnitude. **(C)** Similar rank correlation distribution plots as shown in Figure 3, but with signal-to-noise and brain volume (upper) or the generative spatial parameter (lower) regressed out. Rank corr: spearman correlation; gen. param: spatial generative modeling parameter.

The MaMI database contains mammals of various brain shapes and sizes, which could influence the comparison between network and phylogenetic distances. The brain volumes of the mammals in the database span four orders of magnitude (Figure 4A). Importantly, we find that the spatial embedding of these brain networks, as estimated by our generative modeling approach, is highly correlated with brain volume (Spearman’s ρ: −0.597, *p* < 10^–6^; Figure 4B). That is, estimating the influence of spatial distances on edge formation, we found that larger brains are more likely to form shorter connections (incur greater distance penalties), as indicated by a steeper spatial power law term (more negative value of η) estimated *via* a generative modeling framework (Betzel et al., 2016). These two features, brain volume and the spatial generative modeling parameter η, in addition to scan signal-to-noise ratio (Supplementary Figure 5), were used as covariates of no interest for computing a partial correlation between brain network and phylogenetic distances (Figure 4C). These features were compared in a pairwise manner by taking the geometric mean between each pair of data points. This way, the data could be averaged within blocks and used as covariates with the same dimensionality as the distance measures. Note that the geometric averaging of per-mammal features makes for an unconventional partial correlation, necessitated by the need to match the dimensionality of the pairwise distance data. As before, brain network distances computed with the Laplacian spectrum displayed correlations with the largest positive magnitudes, with the five-dimension Laplacian spectral distance again displaying the highest magnitudes. When co-varying for the spatial bias, the distributions formed by Jensen–Shannon divergence of the Laplacian spectrum and the binary NetSimile distance fail to clear the zero threshold and are thus colored gray in Figure 4C. In Supplementary Figure 11, we demonstrate the effect of using just signal-to-noise ratio as a covariate of no interest. Finally, in Supplementary Figures 8–10, we visualize correlation distributions taken by comparing the network and phylogenetic distances at the level of edges, without averaging into block-wise values. In this case, correlation coefficients are attenuated, which could be due to measurement or estimation uncertainty at the edge-level. However, many measures’ distributions still exceed the zero threshold, even after considering the partial covariates of signal-to-noise ratio, brain volume, and geometry. Furthermore, we demonstrate that the choice of distance model used to operationalize spatial embeddedness does not fundamentally change the results of these analyses. For the purposes of estimating a rank ordering of spatial embeddedness across the mammalian brains of the MaMI database, the two models converge to highly similar trends (Supplementary Figure 7). Using parameters based on an exponential distance rule, we replicate the brain network and phylogenetic distance results in Supplementary Figures 12, 13.

**FIGURE 5:**
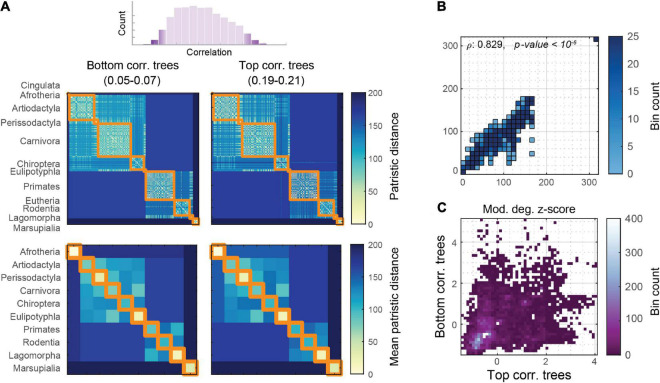
Phylogenetic distances sampled from the top and bottom of the correlation distribution. **(A)** Average phylogenetic distances, at mammal level (upper) and taxonomic block (lower) from both the top 1% and bottom 1% of the rank correlation distribution of the network portrait divergence. **(B)** Correlation between the top and bottom phylogenetic distance matrices, showing generally strong rank correlation. **(C)** Within-module degree z-score between the top and bottom phylogenetic distance matrices, showing that lower z-scores (below zero) are generally similar, whereas larger z-scores have a greater spread.

We can inspect the upper and lower tails of the network portrait divergence correlation distribution to identify and describe phylogenetic trees that both strongly and weakly covary with brain network distances. To what extent would these trees look different? To answer this question, the trees generating both the top and bottom 1% of correlation distribution were collated, corresponding to correlation magnitude ranges of 0.05–0.07 and 0.19–0.21, respectively. These distance matrices were averaged to visualize the extent to which phylogenetic distances correlate with brain network distances (Figure 5A). The edge-level entries between these matrices are highly correlated (Spearman’s ρ: 0.829, *p* < 10^–6^; Figure 5B), despite being derived from the extreme ends of the correlation distribution. By comparing the within-module degree z-score distributions of these phylogenetic distance matrices (using the taxonomy as the partition), we can visualize how differences are expressed at higher z-scores (i.e., larger distance magnitudes; Figure 5C).

## Discussion

Here we study the difference in the connectional topology of brains from a large sample of mammalian species, and we demonstrate that distances between topologies relate to both grouping of species into taxonomic orders and their phylogenetic distances. Our central finding is that differences in connectome-derived topological features are related to distances between species derived from homologies in genetic sequences, collated into a phylogenetic tree (Figure 1). These findings are supported by applying numerous network distance measures and exploring networks at different density thresholds. Importantly, we show that the relationship between network and phylogenetic distance holds after accounting for several potentially confounding variables.

Our work builds on and extends previous empirical studies that have compared features of mammalian brain architecture and connectivity. Previous studies have identified the presence of similar areas and large-scale networks across species exemplars (Lu et al., 2012; Balsters et al., 2020) and have compared their network properties (Miranda-Dominguez et al., 2014). Across exemplar networks from a mouse, fly, macaque, and human, a consistent weight-to-distance relationship was identified, which was further probed to suggest that long distance connections across these brains increases functional diversity (Betzel and Bassett, 2018). Several aspects of our work go beyond prior literature. While previous studies have largely focused on small sets of “model organisms” we perform a comparative analysis across many species and specimens (Assaf et al., 2020; Suárez et al., 2022). Whereas previous work on this database primarily establishes topological commonalities between mammals, such as similar shortest path length topologies and the preservation of distance-dependent connectivity strength, the work here focuses on the pattern of distances formed when comparing the mammals in a pairwise manner.

Using network portrait divergence as a starting point, we demonstrate that the distance within taxonomic order is smaller than between orders (Figures 2B, C). Network portrait divergence is derived using shortest paths (i.e., geodesic distances), which can be conceptualized as a proxy for network communication processes (Goñi et al., 2014). Therefore, we could speculate that mammals within an order have brain network topologies that support communication processes to a similar extent. We additionally examine this effect using other network distances that focus on the graph spectra or other feature distributions (Figure 2D). Between-mammal network distance patterns are then correlated with between-mammal phylogenetic distance patterns (Figure 3), demonstrating a link between brain networks and phylogenetic information. Notably, we observe non-zero correlations for many of the distance measures, which index a variety of network characteristics. For example, the low-dimensional Laplacian spectra carry information about a network’s community structure (de Lange et al., 2014). The observation that the Laplacian spectral distance relates the most to phylogenetic distance could indicate the importance of community structure as a useful network feature to discriminate between mammalian brain topologies. Since these relationships hold across many distance measures, we can feel more confident that these results are not an artifact of a single feature. Additionally, these relations persist when potential confounding factors, including scanning signal-to-noise ratio, brain volume and geometry, are taken into account (Figure 4). When replicating these analyses at the level of edges, these relationships are attenuated, possibly indicating the possible noisiness of making these comparisons at a more fine-grained level. This scale of analysis could be the focus of future work, especially considering that the difference between top and bottom-correlating trees might be most apparent at the within-taxonomic order level (Figure 5C). Additional future work is needed to further identify the underlying factors that likely drive these findings. Possible candidates include factors that capture the different body plans, sensory and motor adaptations, behavioral capacities, and ecological contexts of each species.

One of the most important factors influencing the layout of anatomical/structural connections is the spatial separation between brain regions. Virtually all previous studies of connection topologies, including studies carried out with histological techniques such as tract tracing (Markov et al., 2014; Coletta et al., 2020), have demonstrated that connection density varies with the physical distance between neurons (intra-regionally) (Ercsey-Ravasz et al., 2013; Horvat et al., 2016) and between segregated cortical regions (Betzel et al., 2017). Consistent with prior studies carried out on just a small sample of model organisms, we find that a generative model applied to brains from a wide range of mammalian different species varies systematically with brain size. This finding supports the notion that, as brain get bigger, connectivity becomes progressively more expensive (Ringo et al., 1994) as the cost of wiring (edges) increases faster than the expansion of gray matter (nodes). The resulting drop in the density of long-distance connections presents an increasingly severe challenge to communication and information integration (Laughlin and Sejnowski, 2003; Bullmore and Sporns, 2012). This challenge merits future exploration and investigation. For example, one universal aspect of mammalian cortical topology is the existence of network communities, or modules, interlinked by long-distance paths and highly connected hubs (Harriger et al., 2012; Zamora-Lopez et al., 2016; Faskowitz and Sporns, 2020; Liu et al., 2020; Swanson et al., 2020). Our approach can be extended to leverage the detection of modules across different species and taxa. Another future direction is the use of various measures of network communication (Seguin et al., 2020) to investigate possible differences in efficient communication strategies across species.

### Limitations and future directions

A fundamental challenge of comparative studies is the necessity to match data derived from brains with differing shapes, sizes, and overall morphology. Although a brain network modeling approach is seemingly preferable for these comparisons, there remain notable limitations. Establishing regional neural correspondence across species and evolutionary branching is a longstanding research question (Krubitzer, 2009; Kaas, 2011; Faunes et al., 2015), with new methods emerging to identify homologies by computationally matching anatomical patterns or fingerprints (Mars et al., 2018; Balsters et al., 2020; Schaeffer et al., 2020; Warrington et al., 2022). In the future, major progress will come from the creation of a universal (pan-mammalian) parcellation or atlas covering the entire cerebral cortex, and ideally extending to subcortical regions as well, a challenge that was first anticipated and initiated by Swanson and Bota (2010). Our current strategy to meet this challenge, which involves the comparison of stochastically generated parcellations (i.e., nodes) between mammals, unfortunately also affects the interpretation of edge weights and correspondence as well (Faskowitz et al., 2022). Thus, we currently cannot precisely localize neuroanatomical structures that drive differences in brain network distances. The random parcellation framework applied here may not be ideally suited to the underlying data in terms of scale (i.e., number of nodes) or delineation of similar functionally coherent areas across brains (Brett et al., 2002; Passingham et al., 2002). To partially mitigate this problem, we employed a scheme to select the most representative network (i.e., least distant) from multiple realizations of connectivity-based parcellation of each mammal scan.

When analyzing scans performed on such a wide variety of brains, data fidelity should be acknowledged as a limitation when interpreting these results. To begin, it is crucial to understand that MRI is unable to image neurons—only anatomical information at the scale of millimeters voxels can be reconstructed (although sub-voxel neuronal properties can be estimated with specialized MRI techniques; Assaf et al. (2008)). The reconstruction of white matter tracts *via* streamline tractography is influenced by spatial embedding (Donahue et al., 2016; Shen et al., 2019; Trinkle et al., 2021) and orientation (e.g., curvature and crossing of fibers) of the anatomy (Jbabdi and Johansen-Berg, 2011). As streamlines are propagated through the white matter, errors can accumulate with length and when encountering complex fiber geometries and multi-direction crossings (Jeurissen et al., 2019). These difficulties are inherent to the inverse problem that tractography aims to solve, but there exist strategies that aim to enhance the anatomical validity of the rendered streamlines. We employ a white matter model that attempts to account for crossing geometry and chose to only retain streamlines that terminate in gray matter (Smith et al., 2012). Additionally, we have further attempted to mitigate the spatial bias at the level of MRI acquisition, by adjusting voxel size to roughly scale with the brain volume for each mammal. Thus, the streamline algorithm should propagate through white matter with a similar number of steps, regardless of the real-world size of the brain. Further validations of the acquisition strategies can be found in Assaf et al. (2020). Streamline weights were normalized by the geometric mean volume of its termination nodes, to account for potential size differences of nodes within the randomly rendered parcellations for each mammal. At the level of network analysis, we employed a common edge-density threshold to demonstrate that the obtained results were not simply byproducts of density differences between species.

The MaMI database presents a unique opportunity to assess brain network commonalities and differences across mammals (Suárez et al., 2022), and to ask questions about what rules might govern the topology of brains across varying sizes and layouts. A key feature of brain networks is their spatial embedding (Bullmore and Sporns, 2012; Stiso and Bassett, 2018), often quantified by models estimating the probability of an edge existing over fiber or Euclidean distance. Spatial embeddedness can be operationalized and modeled with a power law (Betzel et al., 2016) or with an exponential decay function (Ercsey-Ravasz et al., 2013; Oh et al., 2014; Horvat et al., 2016; Theodoni et al., 2022). Here, we adopted both approaches in a computational generative model and we conclude that for the purposes of estimating a rank ordering of spatial embeddedness, the two models converge to highly similar trends using the MaMI data (Supplementary Figure 7). However, the present analyses by themselves do not support the veracity of one model over another. In future work, the MaMI database could serve as a resource for directly comparing spatial embedding models for brain networks. Along these lines, future work could also focus on the estimation of connectivity magnitude (i.e., edge weight), given spatial embeddedness. The present generative modeling analysis is limited to the use of binary brain networks. This approach could be expanded to further model edge weight distributions, such as in the weighted stochastic block model (Faskowitz et al., 2018), which might interact with the spatial embeddedness or position of network nodes. Overall, the present manuscript demonstrates that differential spatial embedding does not completely explain the relationships between brain network and phylogenetic distances. The topic of spatial embedding remains an important area for future research, especially in the context of brain evolution.

## Conclusion

To conclude, we compared brain networks from over 100 unique mammalian species and demonstrate how comparing these networks can relate to both taxonomic divisions and phylogenetic distance between mammals. Not only are within-order distances smaller than between-order distances, but these network distances positively correlate with estimated phylogenetic distances as well. By comparing imaging-derived brain network information and DNA-sequence-based phylogenetic information, our analyses span vastly different data modalities. This study paves the way for future research on linking specific topological configurations with differences in species-level traits.

## Data availability statement

The raw data supporting the conclusions of this article will be made available by the authors, without undue reservation. Code to perform data analyses can be found on GitHub: https://github.com/faskowit/faskowitz2022mammals.

## Ethics statement

No animals were deliberately euthanized for the present study. All brains were collected based on incidental death of animals in zoos in Israel or natural death collected in the wild and with the permission of the national park authority (approval no. 2012/38645), or its equivalent in the relevant countries.

## Author contributions

YY and YA collected and pre-processed the imaging data. JF and MP performed additional data pre-processing and performed network modeling and statistical analysis. JF wrote the first draft of the manuscript. All authors contributed to the conception and design of the study, manuscript revision, read, and approved the submitted version.

## Footnotes

1
vertlife.org/phylosubsets/
