# Supplementary material for: Connectome topology of mammalian brains and its relationship to taxonomy and phylogeny
Source: Front Neurosci. 2023 Jan 11;16:1044372. doi: 10.3389/fnins.2022.1044372 (PMC9874302; doi:10.3389/fnins.2022.1044372)
Supplement: Supplementary file 1 [file Data_Sheet_1.pdf]

Supplementary Information: Connectome topology of mammalian brains and its relationship to taxonomy and phylogeny

Supplementary Table S1 Distance measure abbreviations and descriptions.

Abbreviation	Short description
bin-gen-max	Binary generative model Kolmogorov–Smirnov distance maximum
bin-gen-mean	Binary generative model Kolmogorov–Smirnov distance mean
wei-gen-max	Weighted generative model Kolmogorov–Smirnov distance maximum
wei-gen-mean	Weighted generative model Kolmogorov–Smirnov distance mean
lap-spec-full	Laplacian spectral distance with all eigenvalues
lap-spec-5	Laplacian spectral distance with 5 eigenvalues
lap-spec-50	Laplacian spectral distance with 50 eigenvalues
lap-spec-100	Laplacian spectral distance with 100 eigenvalues
lapspec-js	Jensen-Shannon divergence between spectral distributions
adj-spec-full	Adjacency spectral distance with all eigenvalues
adj-spec-5	Adjacency spectral distance with 5 eigenvalues
adj-spec-50	Adjacency spectral distance with 50 eigenvalues
adj-spec-100	Adjacency spectral distance with 100 eigenvalues
wei-netsimile	NetSimile distance with analogous weighted measures
bin-netsimile	NetSimile distance
netpd	Network portrait divergence

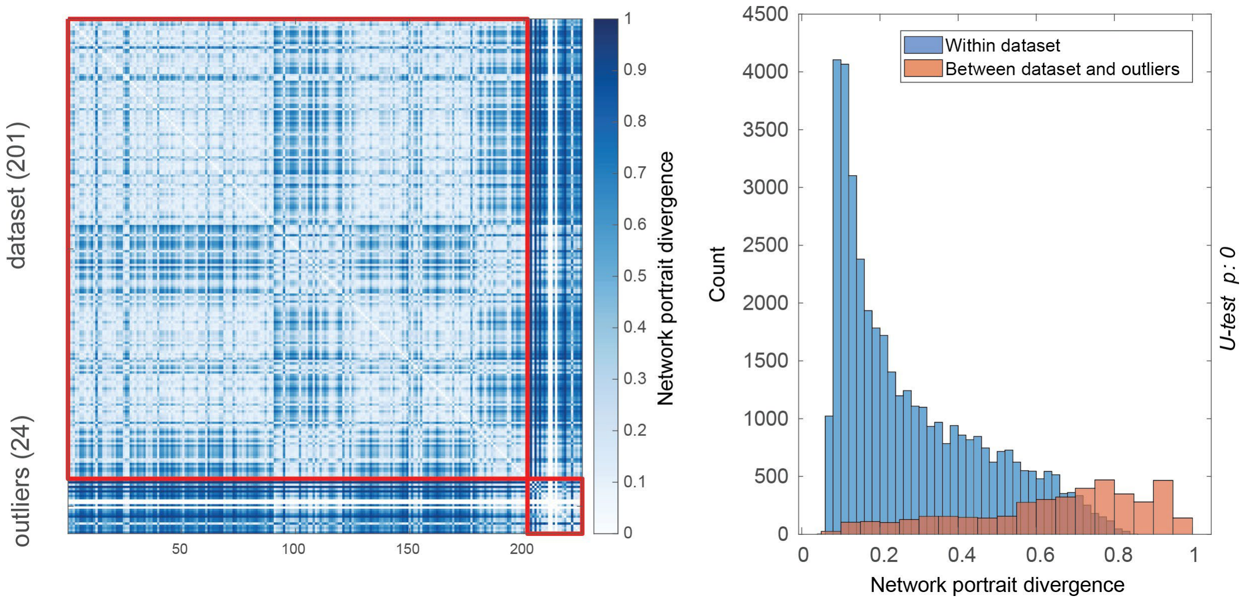

Supplementary Figure S1 Visualization of the main dataset and outliers removed from the original database. On the left, the pairwise network portrait divergence of the 225 mammals, divided by the main dataset and outliers. On the right, the divergence values within the main dataset, and between the main dataset and outliers, presented as a histogram.

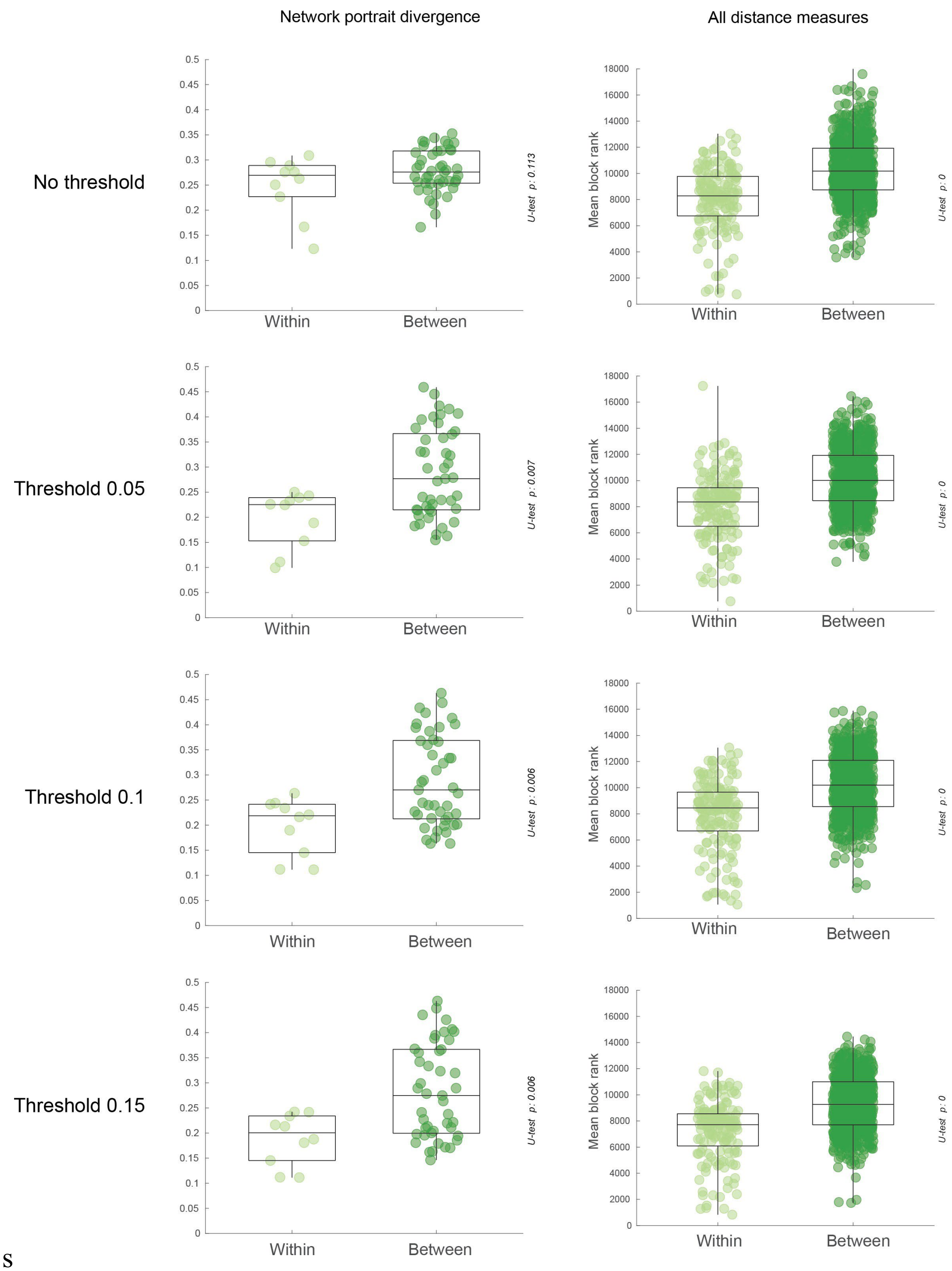

s

Supplementary Figure S2 Within- and between-taxonomy differences across varying thresholds. Different network thresholds (or lack thereof) specify how many edges are retained for each mammalian brain network; first column shows the difference in network portrait divergences, second column shows the rank difference across all network distance measures; non-parametric Mann-Whitney test p-values displayed.

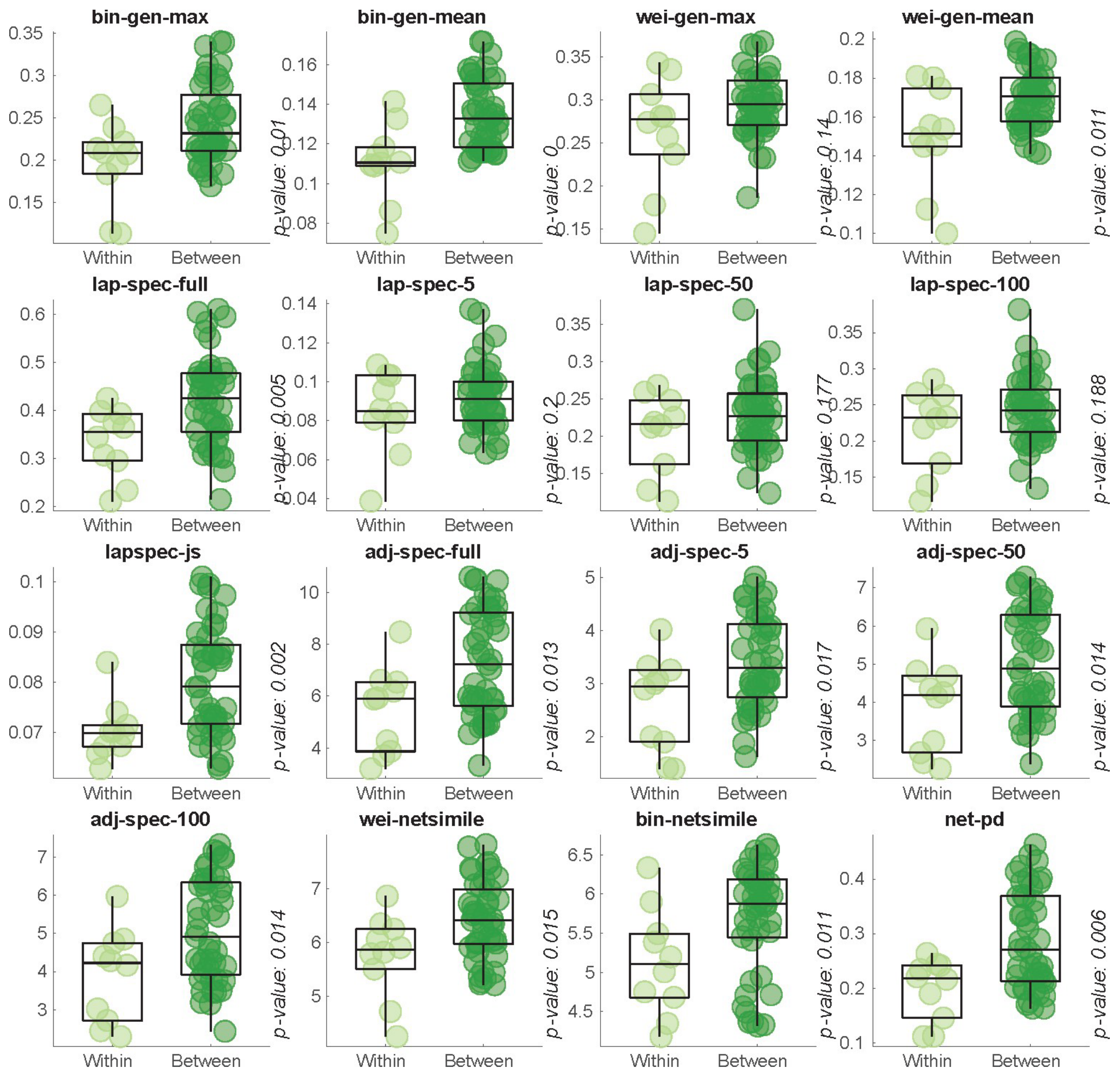

Supplementary Figure S3 Within- versus between-block network distances for all 16 distance measurements, using the 0.10 thresholded data; *p-values* refer to the outcome a Mann-Whitney comparing each pair of distributions; refer to supplementary table S1 for distance abbreviations.

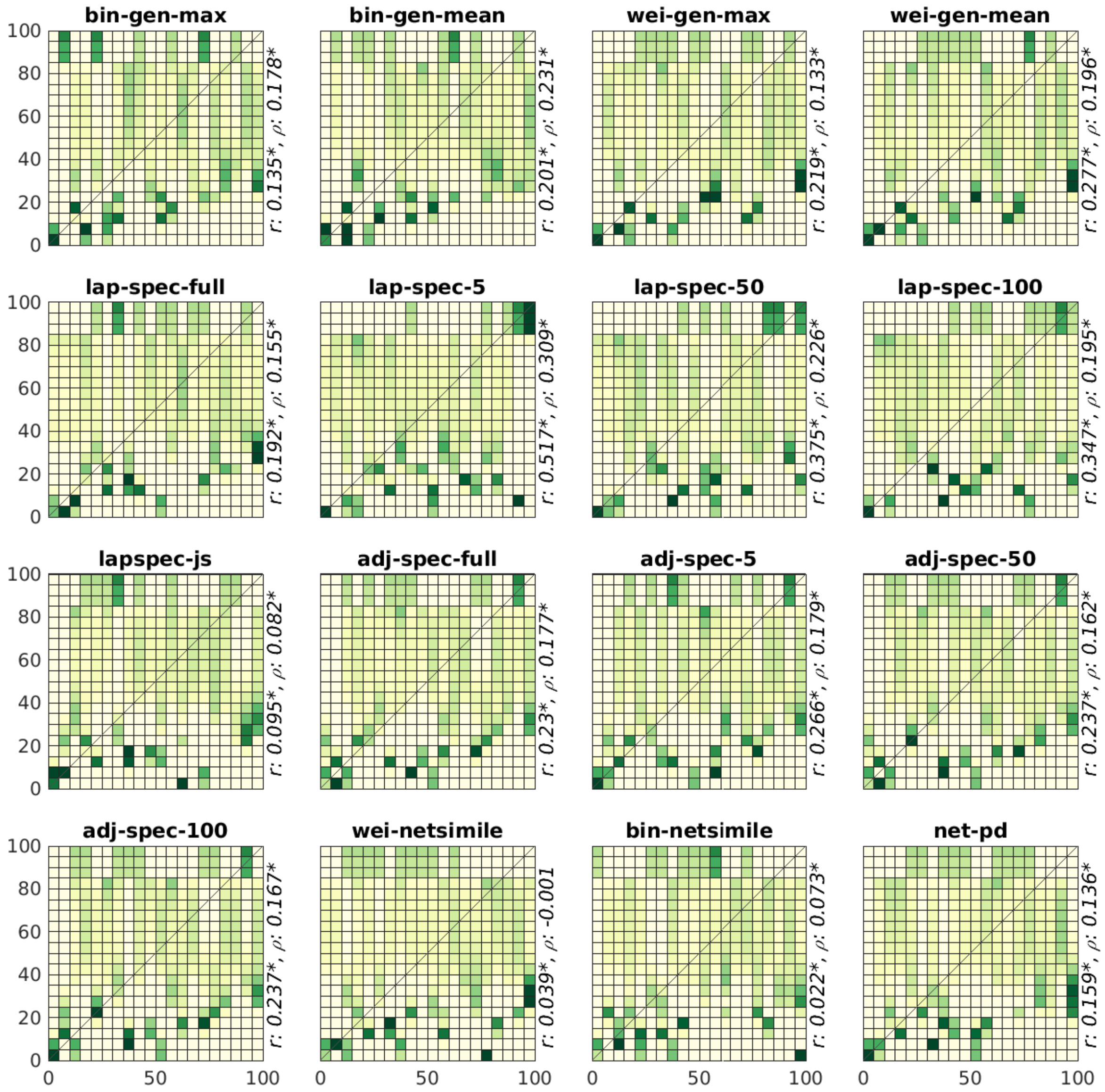

Supplementary Figure S4 2-d histograms of network and phylogenetic distances at the edge level at the 0.1 threshold. For each distance measure, a 2-dimensional histogram shows the relationship between percentile-transformed network distance at 0.1 threshold (x-axis) and the patristic distance (y-axis), across 10^4 phylogenetic trees, at the single mammal level; Pearson and Spearman (rank) correlations are reported; asterisks correspond to correlations with p-values less than 10^{-6}.

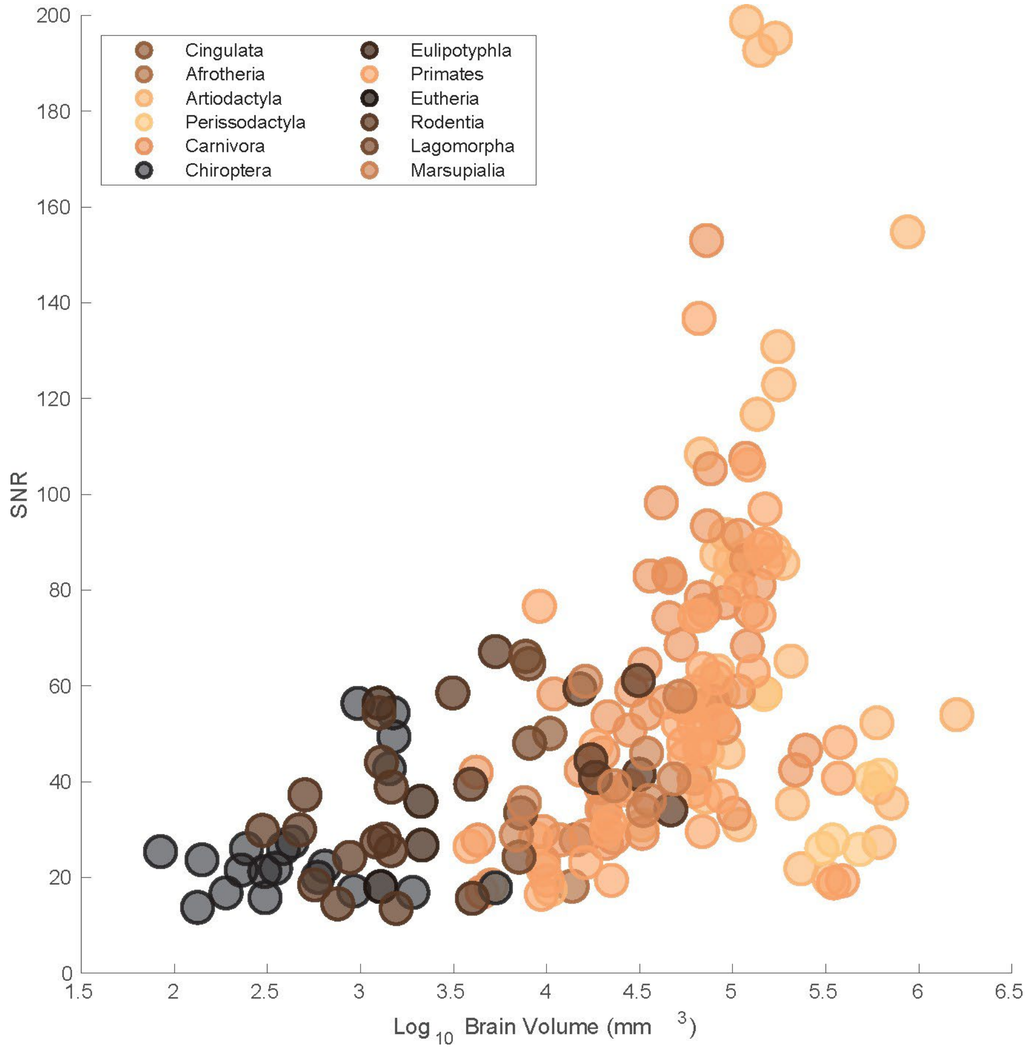

Supplementary Figure S5 Signal to noise ratio (SNR) plotted against brain volume, with mammal orders distinguished by color.

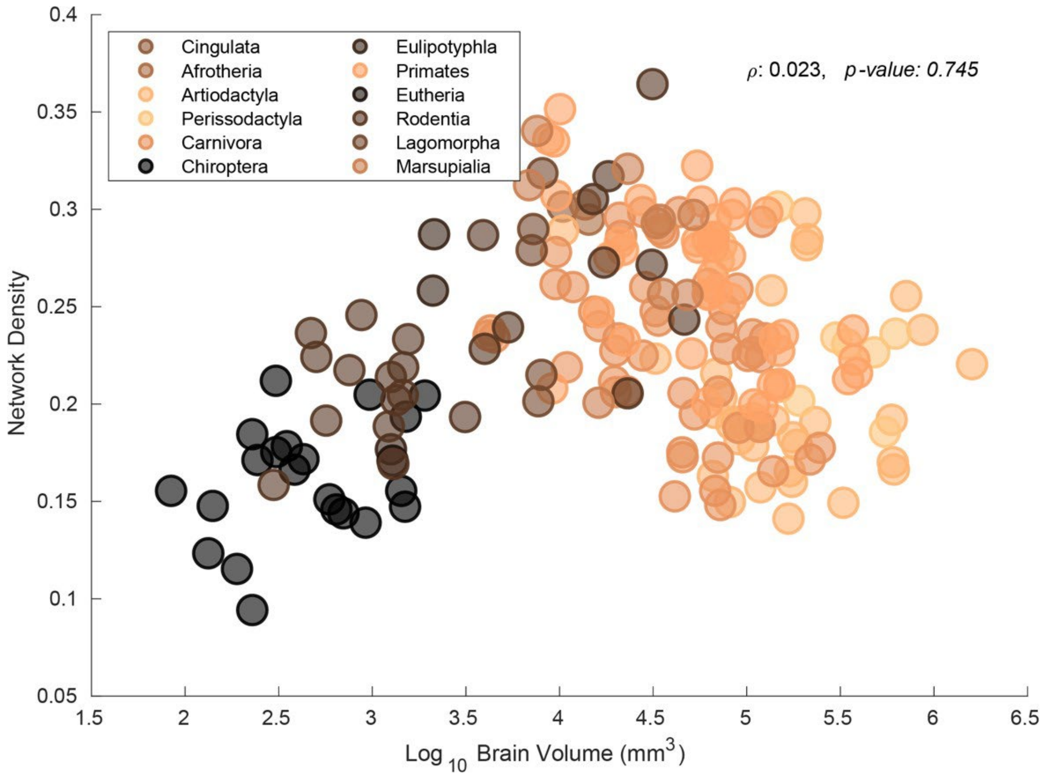

Supplementary Figure S6 Network density plotted against brain volume, with mammal orders distinguished by color.

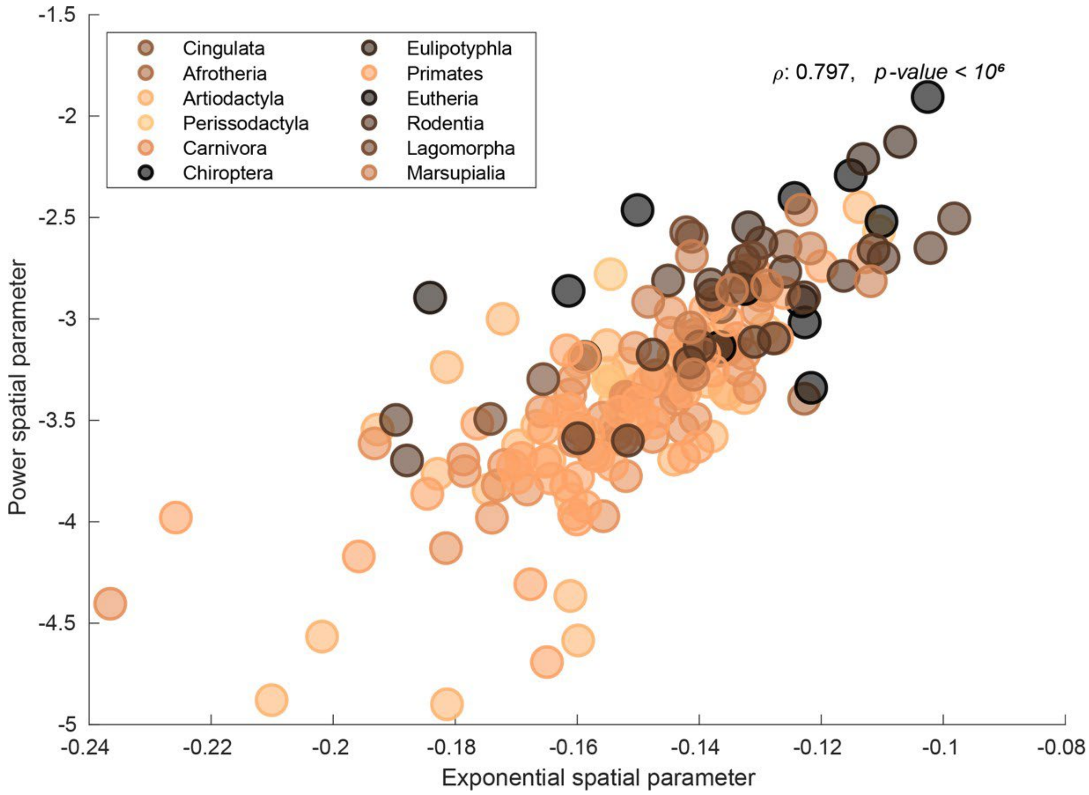

Supplementary Figure S7 Generative modeling parameters from the power-law and exponential models plotted against each other, using the 10% thresholded data, with mammal orders distinguished by color.

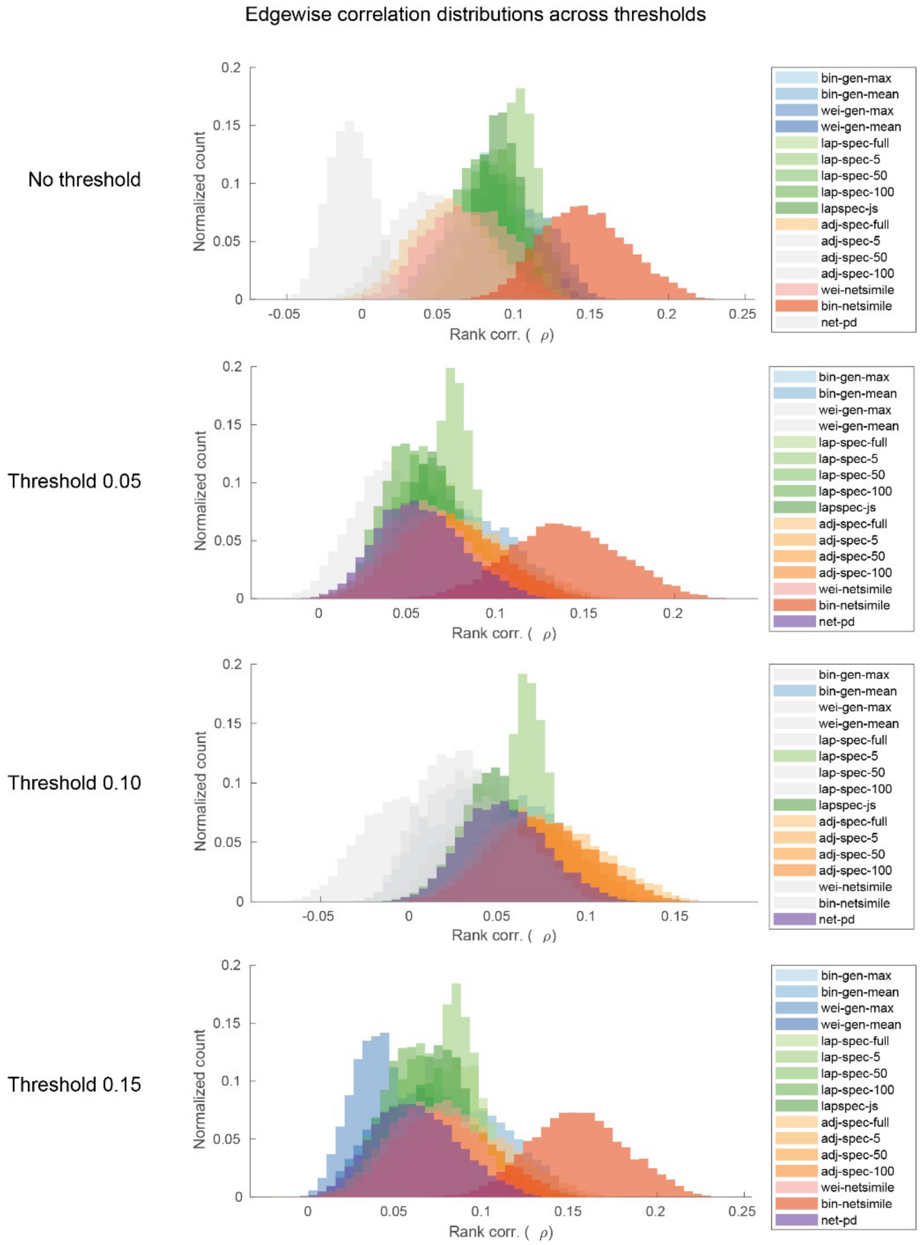

Supplementary Figure S8 Distributions of correlations for each distance measure, where correlations (Spearman) are taken by comparing the network distance and phylogenetic distance at the level of each edge.

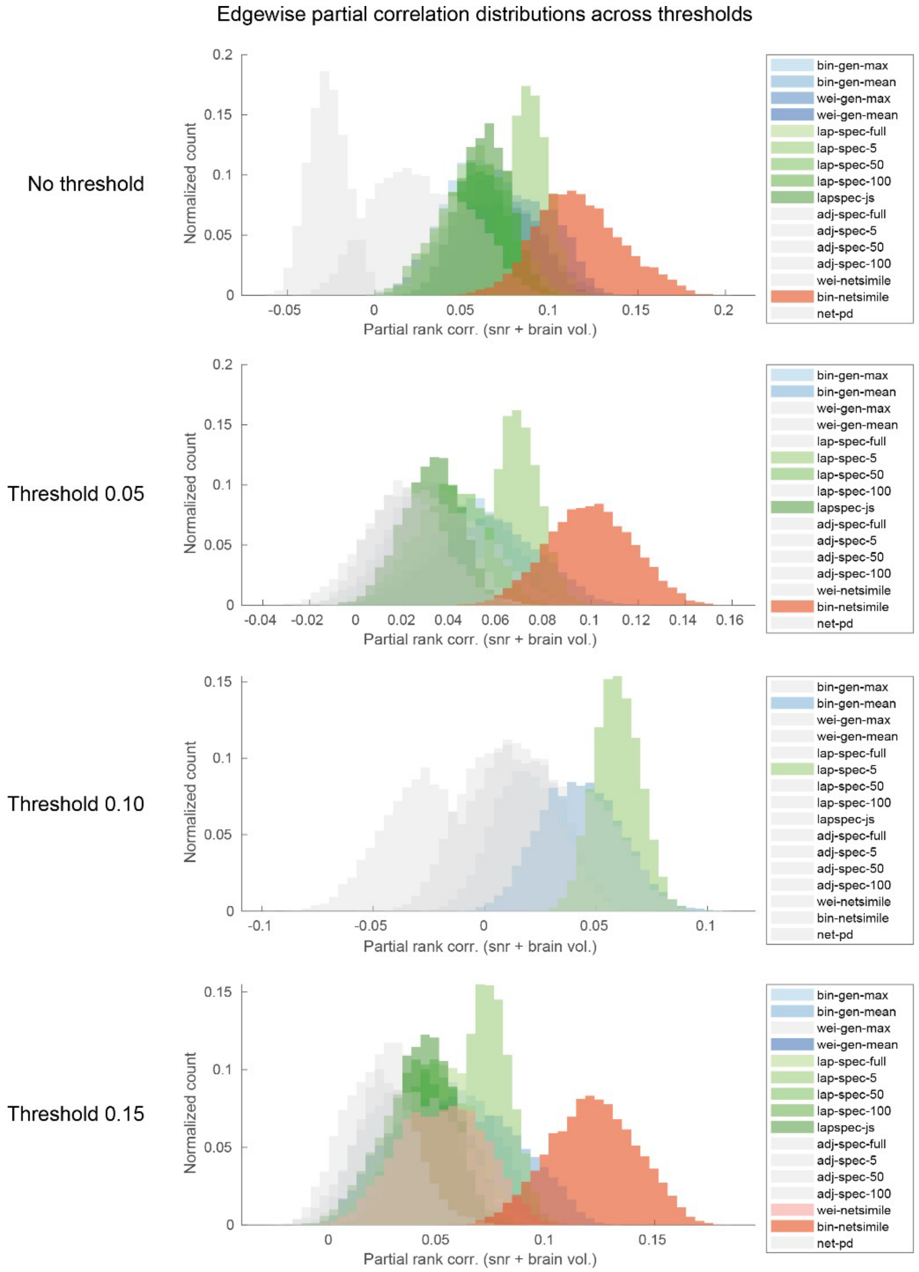

Supplementary Figure S9 Distributions of partial correlations for each distance measure, where correlations (Spearman) are taken by comparing the network distance and phylogenetic distance at the level of each edge; correlations treat signal-to-noise ratio and brain volume as confounding variables of no interest.

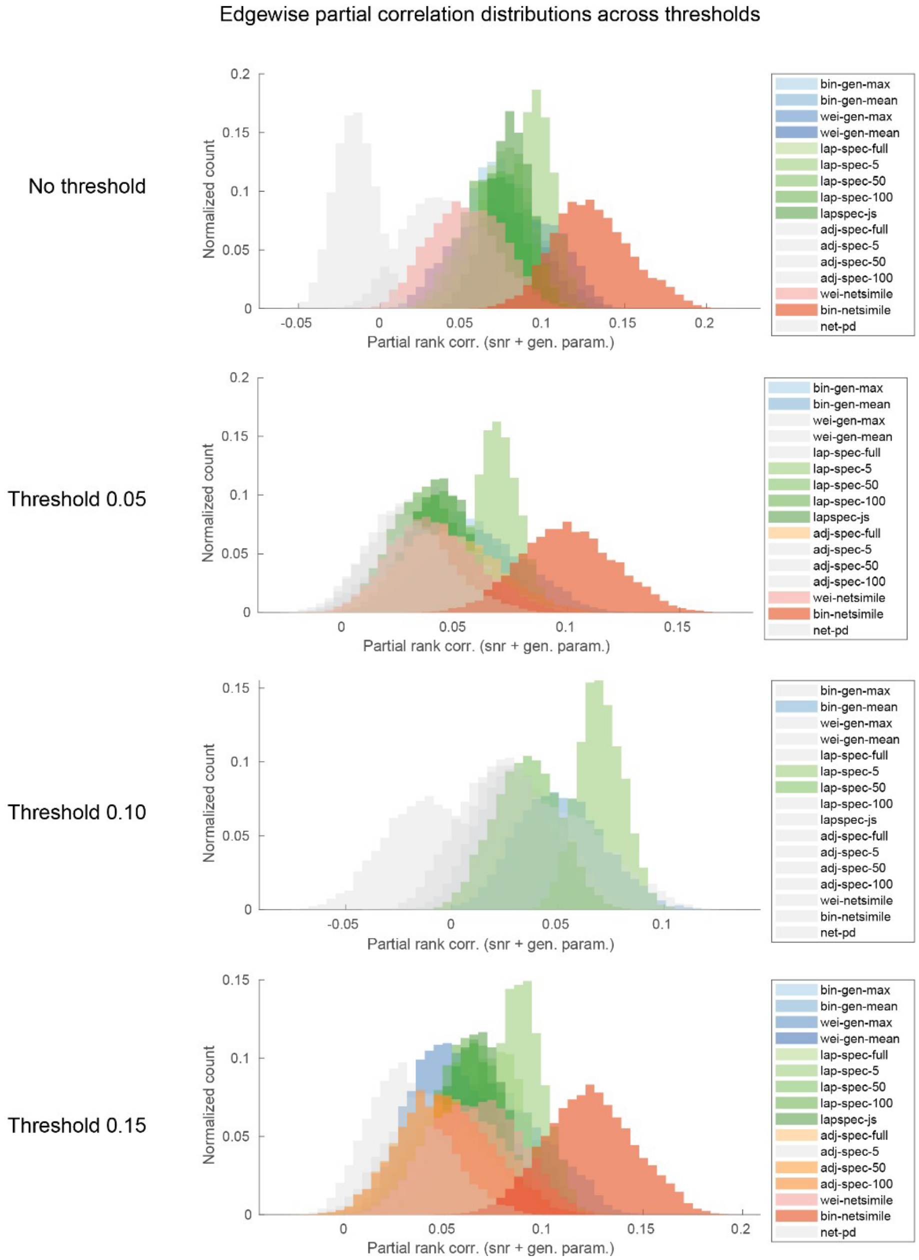

Supplementary Figure S10 Distributions of partial correlations for each distance measure, where correlations (Spearman) are taken by comparing the network distance and phylogenetic distance at the level of each edge; correlations treat signal-to-noise ratio and the power-law spatial generative parameter as confounding variables of no interest.

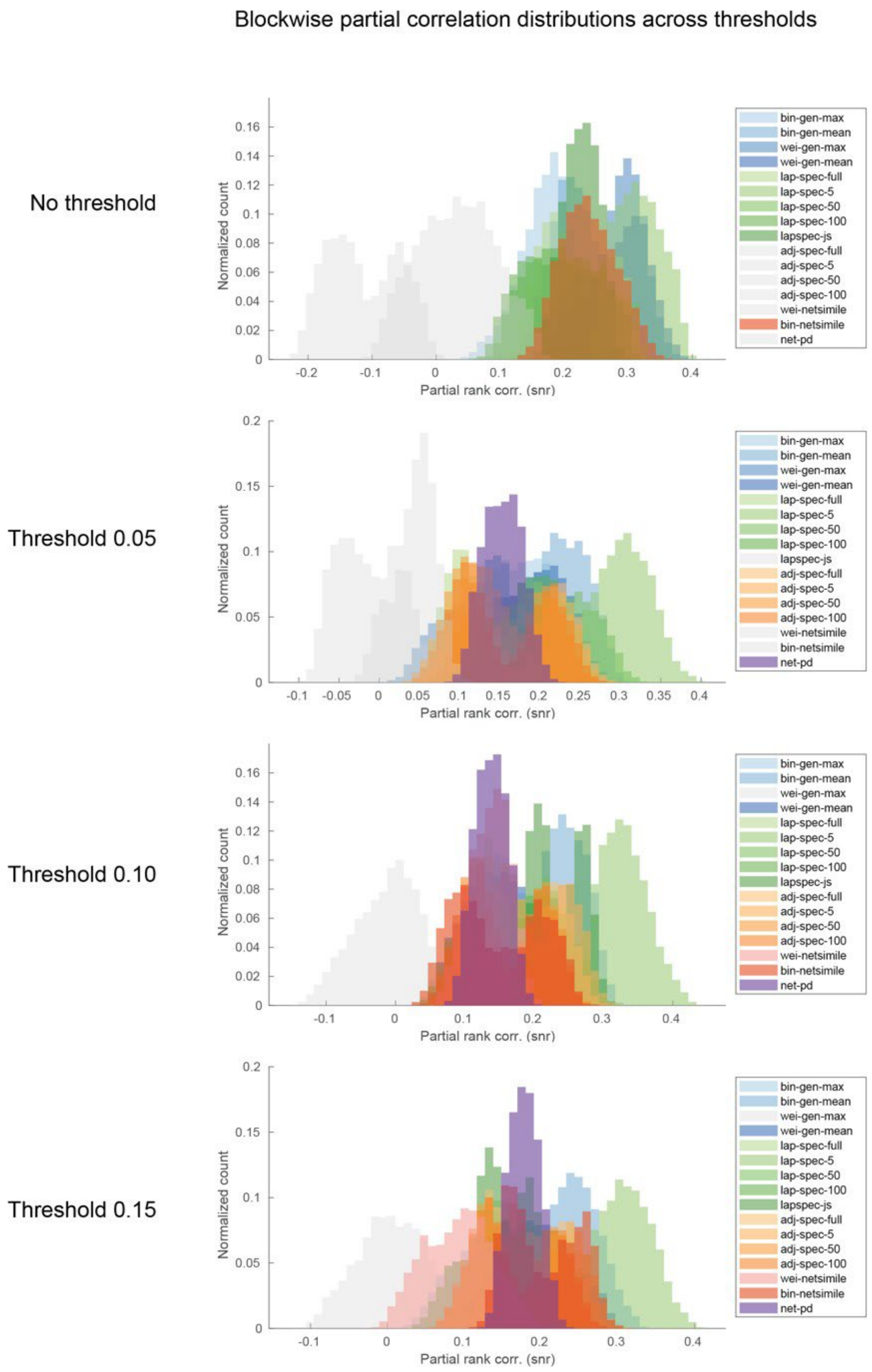

Supplementary Figure S11 Distributions of partial correlations for each distance measure, where correlations (Spearman) are taken by comparing the network distance and phylogenetic distance at the level of blocks; correlations treat signal-to-noise ratio as a confounding variable of no interest.

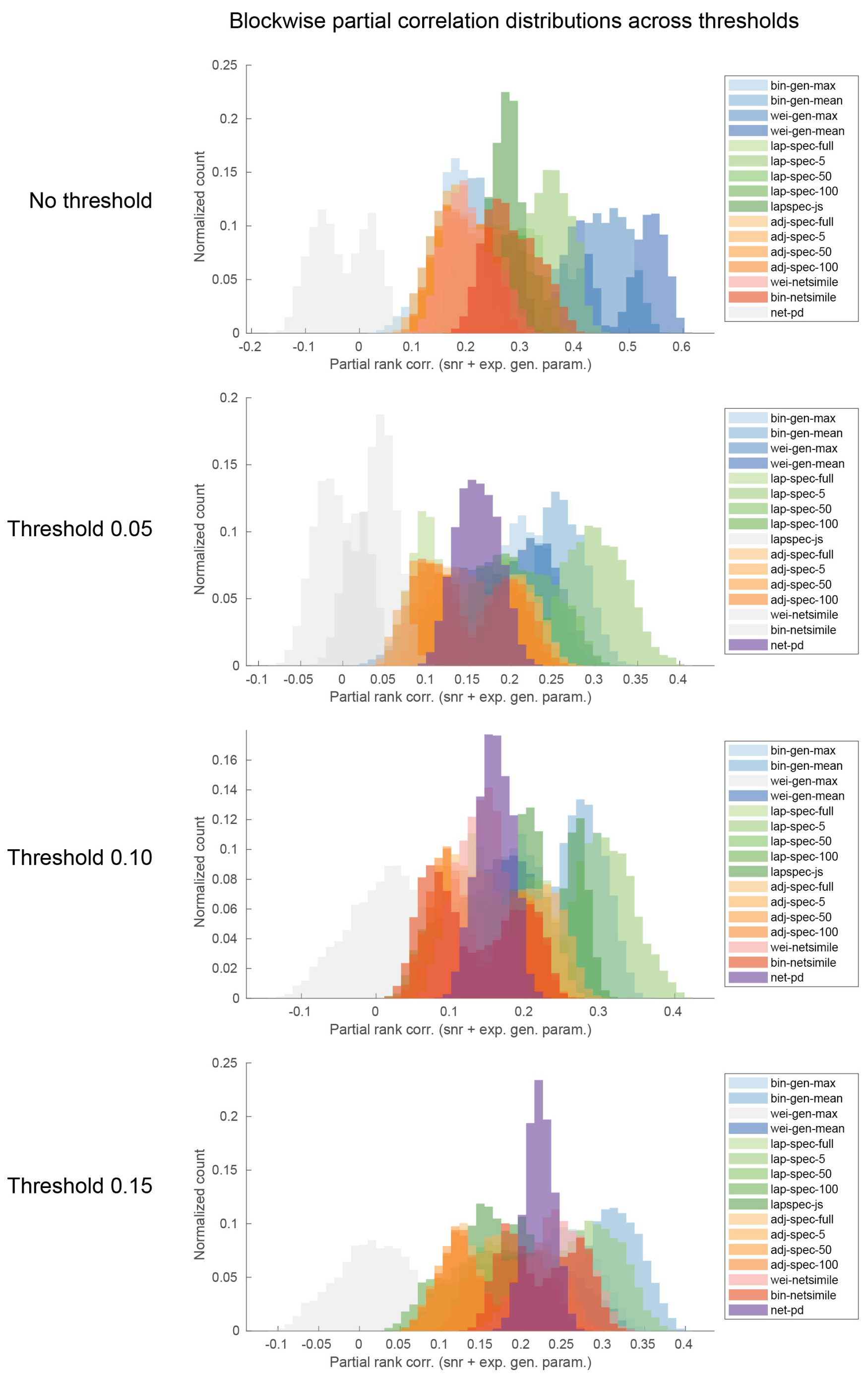

Supplementary Figure S12 Distributions of partial correlations for each distance measure, where correlations (Spearman) are taken by comparing the network distance and phylogenetic distance at the level of each block; correlations treat signal-to-noise ratio and the exponential spatial generative parameter as confounding variables of no interest.

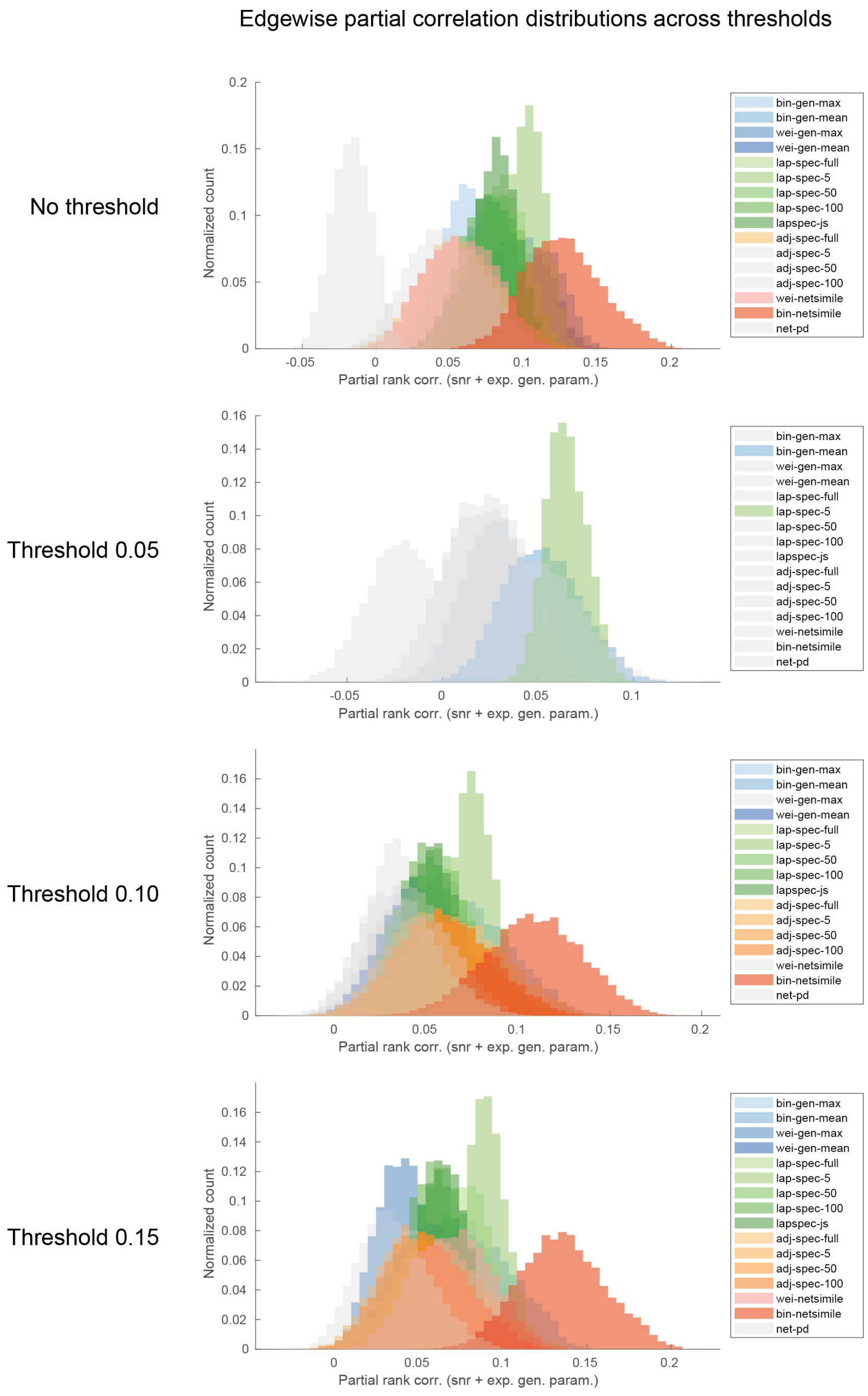

Supplementary Figure S13 Distributions of partial correlations for each distance measure, where correlations (Spearman) are taken by comparing the network distance and phylogenetic distance at the level of each edge; correlations treat signal-to-noise ratio and the exponential spatial generative parameter as confounding variables of no interest.
